# Drug-resistant tuberculosis in Nepal: the sixth national prevalence drug-resistant survey, 2024

**DOI:** 10.1186/s40249-026-01503-z

**Published:** 2026-10-13

**Authors:** Naveen Prakash Shah, Bhuwan Poudel, Prajowl Shrestha, Prasant Vikram Shahi, Lok Raj Joshi, Padmanav Ghimire, Ratna Bahadur Bhattarai, Meera Hada, Thuma Pun, Rajesh Man Rajbhandari, Shraddha Acharya, Bhagwan Maharjan, Rabin Gautam, Barsha Thapa, Kenza Bennani, Bir Bahadur Rawal

**Affiliations:** 1 National Tuberculosis Control Center, Thimi, Bhaktapur Nepal; 2 World Health Organization (WHO), Kathmandu, Nepal; 3 Save the Children, Kathmandu, Nepal; 4 Centre for Molecular Dynamics Nepal, Kathmandu, Nepal

**Keywords:** Rifampicin resistance, Multidrug-resistant, Risk factor, Antimicrobial stewardship, Tuberculosis, Treatment

## Abstract

**Background:**

Tuberculosis (TB) remains a major public health challenge in Nepal, characterized by increasing drug-resistant TB (DR-TB) and constrained surveillance capacity. The sixth national DR-TB prevalence survey was therefore implemented to address this gap. This study aimed to estimate the national prevalence of DR-TB and identify risk factors among new and previously treated pulmonary TB patients.

**Methods:**

A national cross-sectional survey was conducted from December 2022 to January 2024, enrolling bacteriologically confirmed pulmonary TB patients using stratified cluster sampling at 49 diagnostic centers. Phenotypic and genotypic drug sensitivity testing was performed on 1768 *Mycobacterium tuberculosis*-confirmed samples. Logistic regression analysis estimated DR-TB prevalence and its associated risk factors.

**Results:**

Rifampicin resistance was identified in 2.2% [95% confidence interval (*CI*) 1.4–3.6] of new TB patients and 10.1% (95% *CI* 5.1–19) of previously treated patients. In total, 9.4% exhibited resistance to at least one first-line drug, with isoniazid exhibiting the highest resistance rate. Within the rifampicin-resistant TB (RR-TB) group, resistance to moxifloxacin was more prevalent among newly diagnosed patients than previously treated patients at both critical concentrations (55.3% vs. 33.3% at 0.25 µg/ml; 34.2% vs. 11.1% at 1 µg/ml). Previous TB treatment emerged as the strongest predictor of rifampicin resistance [adjusted odds ratio (a*OR*) = 4.1, 95% *CI* 2.6–6.4].

**Conclusions:**

The prevalence of RR/multidrug-resistant (MDR)-TB in Nepal remains stable compared with previous surveys. However, a high rate of fluoroquinolone resistance among new patients underscores the urgent need for improved antibiotic stewardship and expanded drug-susceptibility testing services. These findings provide essential evidence to guide national TB control strategies.

**Supplementary Information:**

The online version contains supplementary material available at https://doi.org/10.1186/s40249-026-01503-z.

## Key messages

- Nepal’s rifampicin-resistant and multidrug-resistant TB prevalence remains stable compared with previous national surveys, indicating sustained effectiveness of TB treatment and control strategies.
- Previous TB treatment is the strongest risk factor for rifampicin resistance, underscoring the importance of treatment adherence, follow-up, and early drug susceptibility testing.
- High fluoroquinolone resistance, specifically among new RR-TB patients, is a growing concern, with implications for the effectiveness of WHO-recommended MDR-TB regimens.
- Expanded access to drug susceptibility testing and strengthened antimicrobial stewardship are urgently needed to guide appropriate treatment and prevent further resistance.

**Supplementary Information:**

The online version contains supplementary material available at https://doi.org/10.1186/s40249-026-01503-z.

## Background

Tuberculosis (TB) remains a significant global public health challenge, despite being preventable and curable. In 2024, an estimated 10.7 million people developed TB, resulting in 1.23 million deaths. This reaffirms TB as the leading infectious cause of mortality [1, 2] worldwide following the COVID-19 pandemic. The emergence and spread of drug-resistant TB (DR-TB) has created further complications and challenges in global TB control efforts. Approximately 390,000 people developed multidrug- resistant or rifampicin-resistant TB, of whom only 42% of these cases received treatment [1].

The World Health Organization (WHO) defines DR-TB as TB caused by a strain of *Mycobacterium tuberculosis* (MTB) that is resistant to at least one tested TB medication [3]. The most common prevalent form is rifampicin-resistant TB (RR-TB). Rifampicin resistance, which when co-occurs with isoniazid resistance, is classified as multidrug-resistant or rifampicin-resistant tuberculosis (MDR-TB/RR-TB).

In Nepal, TB remains a significant public health concern. In 2024, an estimated 67,000 cases were reported, corresponding to an incidence rate of 227 per 100,000 population, and a treatment coverage rate of 60%. The estimated number of TB-related deaths was 15,982, resulting in a mortality rate of 53 per 100,000 population. Nepal is among the top 30 high-burden countries globally for DR-TB, with approximately 1500 cases annually, of which about 42% remain unnotified [1, 4].

Despite persistent challenges, Nepal has expanded WHO-recommended molecular diagnostic services since 2011 to improve TB detection [5]. Continuous surveillance through routine drug susceptibility testing (DST) is the most effective method for systematic monitoring of DR-TB. However, nationwide access to DST remains limited in Nepal. Consequently, the WHO recommends conducting a drug-resistant survey every five years until a comprehensive surveillance system is established. Nepal completed its fifth national drug resistance survey in 2010/2011, which estimated rifampicin resistance at 2.2% in its new cases and 15.4% in previously treated cases [6]. The 10-year interval between surveys has hindered Nepal’s ability to monitor trends in drug resistance and implement evidence-based interventions. Discrepancies between earlier survey findings and recent WHO estimates highlight the urgent need for a sixth national drug-resistant survey. Thus, the present study aims to assess the national prevalence and associated factors of DR-TB.

## Methods

### Survey design and survey population

The study followed the WHO’s fifth edition drug-resistant survey guideline [7]. All newly registered, bacteriologically confirmed pulmonary TB cases in Nepal diagnosed by sputum smear microscopy or Xpert MTB/RIF (Xpert Mycobacterium tuberculosis/Rifampicin assay) were included in the study. Similarly, patients with extra-pulmonary TB, those who had received treatment for more than seven days in the current course, and individuals from settings requiring special administrative clearance, such as prisons or military facilities, were not included in the study.

### Sample design and sample size

A stratified cluster sampling design was employed in which sample size was calculated according to the WHO guidelines. The calculation considered 12,626 confirmed pulmonary TB (PTB) cases with 1% precision, a design effect of 2, and a non-response rate of 10%. The final sample comprised 1724 newly registered PTB patients, and all eligible previously treated patients who provided consent were enrolled (see Additional file 1 for detailed information).

### Sampling strategy and sampling frame

The sampling frame consisted of TB diagnostic centers, stratified by 2020 annual caseloads: Stratum 1 (6–30 cases, 146 sites), Stratum 2 (31–250 cases, 73 sites), and Stratum 3 (> 250 cases, nine sites). Sample sizes were allocated proportionally: 176, 934, and 614 samples were assigned to Strata 1, 2, and 3, respectively. Clusters were independently sampled without replacement in Strata 1 (21 clusters) and 2 (20 clusters), while exhaustive sampling was conducted in Stratum 3. In total, 50 clusters of varying sizes were selected based on logistical, operational, and laboratory processing considerations, including anticipated caseload and transport capacity, to achieve the required sample size (see Additional file 1). One selected cluster did not identify any eligible TB patients during the study period; consequently, 49 clusters were ultimately included in the analysis.

### Data collection tools and techniques

Data collection was carried outfrom December 2022 to January 2024, with intake periods differing across strata as per the sampling design. Standardized face-to-face interviews were conducted using the Clinical Information Form to document sociodemographic, clinical, diagnostic, risk, and treatment information (see Additional file 2). Two barcoded sputum samples per patient were submitted to central laboratories. Patients were registered in both the drug-resistant survey and shipment registers. Field and central teams received training in enrolment, specimen handling, logistics, and laboratory procedures.

### Laboratory procedure

Sputum samples were processed at central laboratories, where they were decontaminated and examined using smear microscopy. Smear-positive samples from the peripheral health facilities underwent GeneXpert testing. All samples were cultured on Lowenstein-Jensen (LJ) solid media. Growth-positive isolates were tested with the MPT64 chromatographic test for rapid identification of the *M. tuberculosis* complex (MTBC). Identified MTBC isolates were subjected to DST using the Mycobacteria Growth Indicator Tube (MGIT) system for phenotypic DST (pDST) and Line Probe Assay (LPA) for genotypic DST. All MTBC isolates underwent first-line DST for isoniazid, rifampicin, and ethambutol. Rifampicin-resistant TB isolates were further tested with second-line DSTfor moxifloxacin, levofloxacin, amikacin, clofazimine, delamanid, bedaquiline, and linezolid. The following drug concentrations were used for the DST: isoniazid (0.1 µg/ml), rifampicin (0.5 µg/ml), ethambutol (5.0 µg/ml), levofloxacin (1.0 µg/ml), moxifloxacin (0.25 and 1.0 µg/ml), amikacin (1.0 µg/ml), clofazimine (1.0 µg/ml), delamanid (0.06 µg/ml), bedaquiline (1.0 µg/ml), and linezolid (1.0 µg/ml).

### Quality assurance

All the procedures adhered to the WHO guidelines [7]. Internal validation of primary variables, treatment history (new or previously treated), was conducted by re-interviewing a randomly selected subset of patients and cross-verifying data across multiple databases. External quality assurance (EQA) included re-testing isolates with discrepant results between pDST and genotypic DST, re-testing 10% of rifampicin-susceptible isolates and all Rifampicin-resistant isolates at alternate central laboratories, and re-testing unresolved cases at the Supranational Reference Laboratory (SRL), Gauting, Germany, for final confirmation (see Additional file 3).

### Data management and analysis

Data were entered into Epi Info (Centers for Disease Control and Prevention, Atlanta, GA, USA) via FortiClient VPN, with periodic quality assurance measures, including double data entry, re-interviews, and cross-verification, to ensure accuracy and reliability. Rifampicin resistance wasassessed using both pDST and LPA results Results from EQA conducted by SRL Gauting were considered definitive.

Data analysis was conducted in R version 4.5.2 (R Foundation for Statistical Computing, Vienna, Austria), following WHO methods [8]. Continues variables were presented as mean ± standard deviation (*SD*) for normally distributed data; categorical variables were presented as frequencies and percentages. Descriptive analyses were performed to examine data distribution, patterns, and trends, with emphasis on patient enrollment, resistance profiles, and determinants of resistance. Logistic regression was applied to estimate the probability of drug resistance and its associations with RR-TB risk factors. Sampling weights were calculated to account for differential enrollment across health-facility strata, defined as the ratio of the expected to the observed number of enrolled new patients within each stratum. All analysesaccounted for the clustered survey design, specifying diagnostic centers as primary sampling units. Weighted logistic regression models with robust standard errors were applied using survey-design-based methods. Statistical significance was set at a two-sided *P*-value of [< 0.05].

Rifampicin Resistance was analyzed using models with and without sampling weights and without multiple imputations for missing data. The model incorporating weight adjustment without imputation was selected, as results were nearly identical across models.

## Results

A total of 2116 patients were enrolled across 49 clusters or sites. Sputum samples from 2111 patients were cultured on LJ media; five samples were excluded due to leakage or unavailability. Among the cultures, 1781 (84.4%) demonstrated growth, 81 (3.8%) were contaminated, and 249 (11.8%) exhibited no growth. Of the cultures with growth, 1768 were identified as MTBC. These 1768 patients, comprising 1592 new cases and 176 previously treated cases, constituted the sample for subsequent analysis (Fig. 1).

**Fig. 1:**
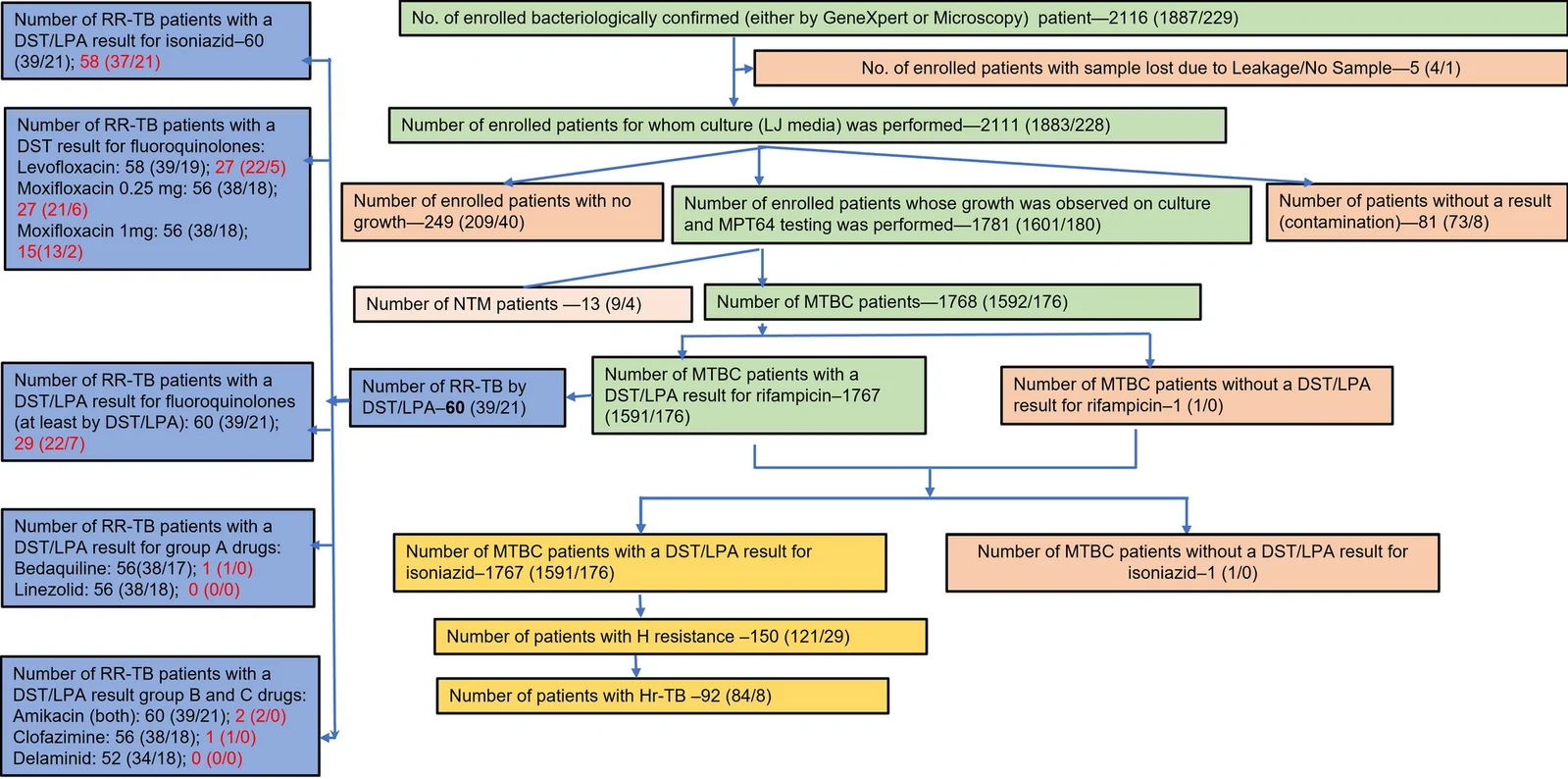
Flow chart of enrolled patients with the test performed and result (considering DST and LPA for rifampicin-resistant). *DST* Drug Susceptibility Testing, *LPA* Line Probe Assay, *LJ* Lowenstein Jensen, *MPT64* Mycobacterium Protein Tuberculosis 64 Antigen, *MTBC* Mycobacterium Tuberculosis Complex, *NTM* Nontuberculous Mycobacterium, *Hr-TB* Isoniazid Mono-resistant Tuberculosis, *RR-TB* Rifampicin-resistant Tuberculosis. Numbers in red font denote the number of resistances, and numbers within parentheses denote the treatment history (New/Previously treated)

### Demographic and clinical characteristics of the participants

Of the enrolled MTBC patients, 70% were male, while the overall mean age was 44 years, with new patients having a mean age of 44 years (*SD*, 19 years) and previously treated patients having a mean age of 45.8 years (*SD*, 17 years). Among new patients, the age group (15–24 years) had the highest representation (20.0%), followed closely by the 65+ age group (19.1%). Among previously treated patients, age distribution was relatively uniform across older age groups, with the 65+ group slightly predominating (18.8%).

HIV status was documented for 862 patients, with five (0.6%) testing positive. Smoking status was available for 1761 patients, of whom 24.5% were identified as current smokers. Diabetes status was reported by 1717 patients (97.1%), with 214 (12.5%) diagnosed with diabetes (Table 1).

**Table 1:** Demographic and clinical characteristics of the enrolled patients

Characteristic	Overall, *N* = 1768^1^ (%)	New patient		Previously treated		*P*-value^3^
		*n* = 1592^1^ (%)	95% *CI*^2^	*n* = 176^1^ (%)	95% *CI*^2^	
Sex						
Female	525 (29.7%)	492 (30.9%)	29%, 33%	33 (18.8%)	13%, 25%	<0.001
Male	1243 (70.3%)	1100 (69.1%)	67%, 71%	143 (81.3%)	75%, 87%	
Age Group						
0–14	27 (1.5%)	27 (1.7%)	1.1%, 2.5%	0 (0.0%)	0.0%, 2.7%	
15–24	343 (19.4%)	318 (20.0%)	18%, 22%	25 (14.2%)	9.6%, 20%	
25–34	265 (15.0%)	235 (14.8%)	13%, 17%	30 (17.0%)	12%, 24%	
35–44	259 (14.6%)	232 (14.6%)	13%, 16%	27 (15.3%)	11%, 22%	
45–54	276 (15.6%)	247 (15.5%)	14%, 17%	29 (16.5%)	11%, 23%	
55–64	261 (14.8%)	229 (14.4%)	13%, 16%	32 (18.2%)	13%, 25%	
65+	337 (19.1%)	304 (19.1%)	17%, 21%	33 (18.8%)	13%, 25%	
HIV Status						
Negative	857 (99.4%)	738 (99.6%)	99%, 100%	119 (98.3%)	94%, 100%	0.15
Positive	5 (0.6%)	3 (0.4%)	0.10%, 1.3%	2 (1.7%)	0.29%, 6.4%	
Unknown	906	851		55		
Current Smoking Status						
Non-Smoker	1330 (75.5%)	1205 (75.8%)	74%, 78%	125 (72.7%)	65%, 79%	0.4
Smoker	431 (24.5%)	384 (24.2%)	22%, 26%	47 (27.3%)	21%, 35%	
Unknown	7	3		4		
Diabetes Status						
Diabetic	214 (12.5%)	188 (12.1%)	11%, 14%	26 (15.4%)	10%, 22%	0.2
Non-Diabetic	1503 (87.5%)	1360 (87.9%)	86%, 89%	143 (84.6%)	78%, 90%	
Unknown	51	44		7		
Strata						
Stratum 1	137 (7.7%)	120 (7.5%)	6.3%, 9.0%	17 (9.7%)	5.9%, 15%	
Stratum 2	766 (43.3%)	724 (45.5%)	43%, 48%	42 (23.9%)	18%, 31%	<0.001
Stratum 3	865 (48.9%)	748 (47.0%)	45%, 49%	117 (66.5%)	59%, 73%	

^1^*n* (%); ^2^*CI* Confidence interval; ^3^Pearson's Chi-squared test and Fisher's exact test

RR-TB was identified in approximately 3.3% of male patients and 3.6% of female patients. The 15–24 years age group contributed the highest proportion of RR-TB cases (18/60, 30%), while the lowest proportion was observed in the 45–54 years age group. No RR-TB cases were reported in the age group of 0–14 years (Fig. 2).

**Fig. 2:**
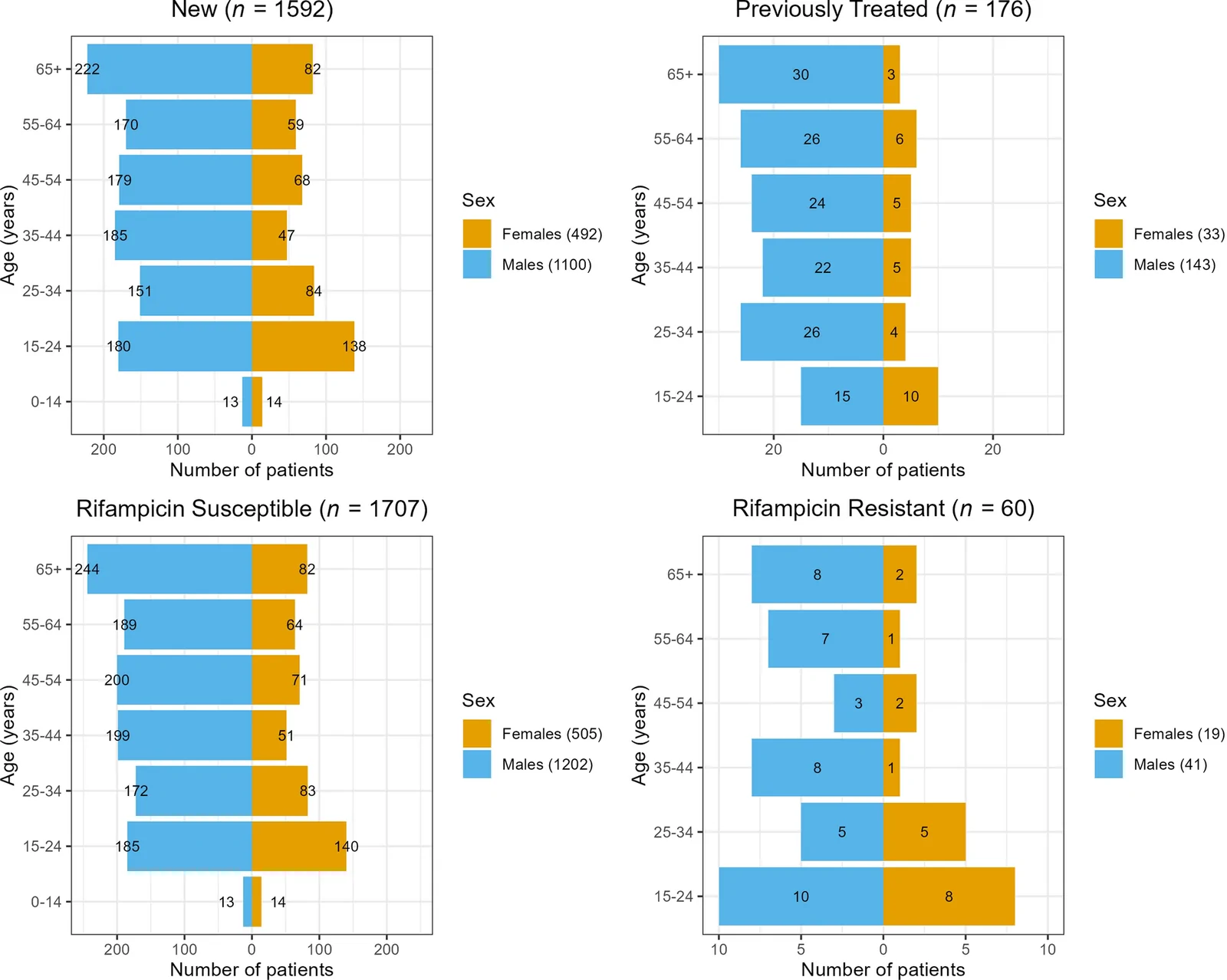
Age-sex distribution of patients by treatment history and rifampicin resistance status. Panels show independent population pyramids for four subgroups: newly diagnosed (*n* = 1592), previously treated (*n* = 176), rifampicin-susceptible (*n* = 1707), and rifampicin-resistant (*n* = 60) patients. Bars represent the number of male (blue) and female (orange) patients within each age group. The x-axis scale differs across panels due to substantial differences in subgroup sample size

### DR-TB prevalence estimates

Of the 1768 patients, 1751 (99.1%) had complete results for all first-line drugs: rifampicin, isoniazid, and ethambutol. In total, 165 patients (9.4%) exhibited resistance to at least one first-line drug, while 1586 (90.6%) were pan-sensitive. The adjusted model showed resistance rates of 8.5% among new patients and 16.4% among previously treated patients. The RR-TB prevalence was estimated at 2.2% for new patients and 10.1% for previously treated patients (Table 2).

**Table 2:** Resistance to any first-line (rifampicin, isoniazid and ethambutol) drugs

Characteristic	Overall	New Patient		Previously Treated		*P*-value^3^
	*N* = 1768^1^	*n* = 1592^1^	95% *CI*^2^	*n* = 176^1^	95% *CI*^2^	
Resistance Status*						
Any Resistant^4^	165 (9.4%)	134 (8.5%)	7.2%, 10%	31 (17.7%)	13%, 24%	<0.001
Pan Sensitive^5^	1586 (90.6%)	1442 (91.5%)	90%, 93%	144 (82.3%)	76%, 87%	
Unknown^6^	17	16		1		

		95% *CI*^2^			
	Prevalence (%)	lower bound (%)		upper bound (%)	
Any Resistance with adjusted model**					
New	8.5	7.1		10.3	
Previously Treated	16.4	11		23.3	
Resistance to rifampicin with the adjusted model					
New	2.2	1.4		3.6	
Previously Treated	10.1	5.1		19	

^2^*CI* Confidence Interval

^3^Pearson's Chi-squared test

^4^Resistant to any of the tested first-line drugs (Rifampicin, Isoniazid, and Ethambutol)

^5^Sensitive to all the tested first-line drugs

^6^ if results for any one of the first-line drugs are missing and no resistance in either of the drugs

*Crude calculation

**Calculated using logistic Regression with clustering and adjusted for strata weight

Isoniazid resistance was more frequent (14.9%) in previously treated patients than in new patients (7.6%). The prevalence of fluoroquinolone resistance (5.2%) and levofloxacin resistance (3.8%) was also greater among previously treated patients. In contrast, resistance to moxifloxacin at both concentrations (0.25 µg and 1 µg) was more prevalent in new patients (55.3% and 34.2%, respectively) than in those previously treated (33.3% and 11.1%, respectively) within the RR-TB group. Bedaquiline resistance was detected in one new patient (2.6%; 95% *CI* 0.3–20) among RR-TB cases. The prevalence of MDR-TB was higher among previously treated patients (10.1%) compared to new patients (2.1%). Similarly, pre-extensively DR-TB was more common in previously treated patients (3.4%) than in new patients (1.3%) (Table 3).

**Table 3:** Resistance pattern of drugs and drug combinations; adjusted for weight and clustering

Drug/Drug combination*	New patient 95% confidence interval**			Previously Treated 95% confidence interval**		
	Prevalence (%)	lower bound	upper bound	Prevalence (%)	lower bound	upper bound
Drugs tested among all the MTBC patients						
Isoniazid	7.6	6.2	9.3	14.9	9.7	22.2
Ethambutol	2.7	1.8	4.0	6.8	3.6	12.7
Fluoroquinolones	2.8	1.8	4.5	5.2	2.3	11.2
Levofloxacin	2.8	1.7	4.4	3.8	1.7	8.5
Drugs tested for those resistant to Rifampicin						
Moxifloxacin 0.25 µg/ml	55.3	33.9	74.8	33.3	12.1	64.5
Moxifloxacin 1.0 µg/ml	34.2	17.5	56.1	11.1	2.3	40.1
Amikacin	5.1	1.2	19.2	0	0	0
Clofazimine	2.6	0.3	20.0	0	0	0
Delaminid	0	0	0	0	0	0
Bedaquiline	2.6	0.3	20.0	0	0	0
Linezolid	0	0	0	0	0	0
Resistance to Drug Combination						
Multidrug-resistant	2.1	1.3	3.5	10.1	5.1	19
Isoniazid mono-resistant (Hr-TB)	5.5	4.5	6.7	4.8	2.2	10.3
Pre-extensively drug resistance	1.3	0.6	2.7	3.4	1.2	8.8
Extensively drug resistance	0	0	0	0	0	0

^*^ For the number tested samples and with results **calculated using logistic regression with robust standard errors to account for clustering

Among RR-TB patients, 48.3% exhibited fluoroquinolone resistance, defined as resistance to levofloxacin or moxifloxacin at 0.25 µg/ml. No cases of resistance to linezolid or extensively drug-resistant tuberculosis (XDR-TB) were found (Table 4).

**Table 4:** Resistance to selected drugs and combinations among rifampicin-resistant tuberculosis

Drug/Drug combination	Number of tested**	Prevalence (%)	95% confidence interval***	
			lower bound	upper bound
Levofloxacin	58	46.6	31.8	62.0
Moxifloxacin (0.25 µg/ml)	56	48.2	33.7	63.0
Moxifloxacin (1 µg/ml)	56	26.8	15.4	42.5
Fluoroquinolones*	60	48.3	35.2	61.7
Bedaquiline	56	1.8	0.2	15.1
Linezolid	56	0	0	0
Pre-extensively drug-resistant	60	48.3	35.2	61.7
Extensively drug-resistant	56	0	0	0

^*^Combination result of levofloxacin, moxifloxacin 0.25 µg/ml, and moxifloxacin 1.0 µg/ml; **Some drugs do not have results for all 60 tested samples because drug susceptibility testing was not performed on certain specimens due to contamination. **calculated using logistic regression with robust standard errors to account for clustering

### Factors associated with RR-TB

The history of previous TB treatment was identified as a significant risk factor for rifampicin resistance, with an adjusted odds ratio *(aOR*) of 4.1 (95% *CI* 2.6–6.4). In addition, patients in Stratum 3, which representsfacilities with large caseloads, exhibited higher odds of RR-TB (*aOR* = 7.3, 95% *CI* 1.3–40.3). Conversely, sex, current smoking status, and diabetes were not found to be significant risk factors (Table 5).

**Table 5:** Risk factors for the development of rifampicin-resistant TB: multivariate analysis

Variables	Total observations^#^	RR-TB *n* (%)^±^	Adjusted Odds ratio	95% confidence interval*		*P*-value**
				lower bound	upper bound	
Treatment history						<0.001
New	1545	39 (2.5%)				
Previously treated	167	19 (11.4%)	4.1	2.6	6.4	
Sex						0.2
Female	504	19 (3.8%)				
Male	1208	39 (3.2%)	0.8	0.5	1.1	
Current smoker						0.7
No	1287	44 (3.4%)				
Yes	425	14 (3.3%)	0.9	0.4	1.7	
Diabetes						0.7
Diabetic	213	8 (3.8%)				
Non-Diabetic	1499	50 (3.3%)	1.1	0.6	2.2	
Strata^†^						
Stratum 1	135	1 (0.7%)				
Stratum 2	748	13 (1.7%)	2.7	0.4	18.5	0.3
Stratum 3	829	44 (5.3%)	7.3	1.3	40.3	0.02

^†^Stratum 1 (small caseload facilities), Stratum 2 (medium caseload facilities), Stratum 3 (large caseload facilities); ^#^Excludes MTB negative patients and those without a rifampicin result; ^±^Percentages represents the proportion of RR-TB cases within each category (row-wise), calculated as n/Total observation for that row; *Calculated using logistic Regression with robust standard errors to account for clustering; **Wald’s test

## Discussion

The survey identified a higher number of male patients than female patients, which aligns with routine data from the National Tuberculosis Program (NTP) [9] and reflects the global epidemiological trend of a higher TB burden among males. This disparity can be attributed to biological predisposition, increased occupational exposure, and differences in health-seeking behaviours. In many low-and middle-income regions, males oftendelay-seeking care due to work-related commitments or stigma-related barriers [10–12]. The predominance of males in Nepal may also result from the strata’s demographics, as large urban health centers were included in the survey and working-age males constitute a significant proportion of the TB caseload.

The estimated prevalence of RR-TB was 2.2% among new patients and 10.1% (95% *CI* 5.1–19) among previously treated patients; these figures are comparable to the2010/11 survey for new patients [6]. The current prevalence among new patients remained unchanged from the previous survey. Although this stability may initially appear encouraging, it warrants cautious interpretation. This suggests that the NTP in Nepal has successfully prevented a major escalation of primary drug-resistant transmission during these periods, reflecting long-term improvements in case detection, directly observed treatment, short-course (DOTS) coverage, and treatment success rates [9]. However, the persistent prevalence also indicates that the program has not yet achieved a substantial reduction in the resistance burden, suggesting ongoing transmission of resistant strains at baseline levels. These results are consistent with data from India, where rifampicin resistance was reported at 2.8% among new patients and 11.7% among previously treated patients [13]. Collectively, these findings suggest that improvements in case management alone are not sufficient to reduce resistance, underscoring the need for more efforts on active case identification and infection control measures.

The overall prevalence of MDR-TB among new patients in Nepal was 2.1%, which is comparable to the WHO South-East Asia Regional estimate of 3.6% for MDR/RR-TB. This similarity indicates that Nepal's resistance profile aligns with regional patterns and does not represent an outlier [14]. The higher prevalence of MDR-TB among previously treated patients, relative to new patients, is consistent with observations from Bangladesh [15], Mongolia [16], Vietnam [17], Thailand [18], and other countries [19–23].

The MDR-TB prevalence among previously treated patients declined from 15.4% (unpublished) in 2011 to 10.1% in this study. However, drug resistance surveys are not designed to provide precise estimates for previously treated patients, resulting in wide confidence intervals. The overlap in confidence intervals necessitates cautious interpretation of the observed trends [18].

The current study findings indicated that 9.4% of patients had resistance to at least one first-line drug (isoniazid, rifampicin, or ethambutol). Higher resistance rates were observed among previously treated patients compared to new patients (16.4% versus 8.5%), indicating that repeated or inadequate treatment likely contributes to the accumulation of drug resistance over time. Among new patients, the prevalence of resistance to any first-line drug (8.5%) was consistent with the fifth national survey results (8.8%, unpublished). Similar observations were reported from Lao PDR (8.4%) [22], which remained higher than in Tanzania (2.1%) [23]. Across all studies, previously treated patients consistently demonstrated greater resistance to at least one first-line drug than new patients [19, 22, 23]. These findings underscore the importance of universal DST at the initiation of re-treatment, a recommendation adopted by Nepal’s NTP that requires stronger enforcement and ongoing monitoring of implementation coverage [6, 24].

The study revealed isoniazid resistance as the most prevalent resistance, which was identified in 7.6% of new patients and 14.9% of previously treated patients. Comparable patterns have been reported from India [25], Lao PDR [22], and Thailand [18]. The substantial burden of isoniazid mono-resistant tuberculosis (Hr-TB) among new patients is significant from a public health standpoint. Hr-TB is often underdiagnosed because standard Xpert MTB/RIF testing does not detect isoniazid resistance, resulting in some Hr-TB patients receiving standard first-line regimens that lack sufficient isoniazid efficacy [26]. These rates are lower than those reported in Thailand but slightly higher than those observed in a study from Peru (new 3%; previously treated 3%) [19].

A single case of bedaquiline resistance was identified in a new patient, indicating possible primary transmission of extensively drug-resistant tuberculosis strains. This scenario poses significant challenges for the long-term effectiveness of newer TB treatment regimens in Nepal. Among patients with RR-TB, moxifloxacin resistance was more prevalent among new patients than among those previously treated (0.25 µg/ml: 55.3% vs. 33.3%; 1. µg/ml: 34.2% vs. 11.1%). The elevated rate of moxifloxacin resistance, especially among new patients, may reflect inappropriate use of fluoroquinolones [27] and the use of antimicrobials in food-producing animals [28]. Nepal has expanded implementation of the WHO recommended BPaLM regimen, comprising bedaquiline, pretomanid, linezolid, and moxifloxacin (BPaLM), for the treatment of MDR/RR-TB. While this regimen offers shorter, more effective treatment options and enhances patient adherence, the rising background prevalence of moxifloxacin resistance in the country raises the possibility that patients initiating BPaLM may already harbour moxifloxacin-resistant strains—potentially compromising the regimen’s efficiency and long-term viability. The findings of the current study strongly support the integration of routine pre-treatment fluoroquinolone susceptibility testing into Nepal’s MDR-TB diagnostic protocols and the strengthening of antimicrobial stewardship in both human and agricultural health [29].

Past treatment history and stratum were the only factors that showed statistically significant association with RR-TB. The significance of the stratum likely reflects the referral patterns of large tertiary health centres in Stratum 3, where retreatment patients tend to concentrate, leading to higher RR-TB detection rates (*aOR* = 7.3, 95% *CI* 1.3–40.3). However, the wide confidence interval associated with this finding warrants cautious interpretation, as it reflects the small number of RR-TB cases in the reference stratum (Stratum 1, *n* = 1) and suggests limited precision in this estimate. This should therefore be considered exploratory, and future surveys with larger stratum-specific sample sizes would be needed to confirm this association. Previously treated patients exhibited 4.1 times higher odds of RR-TB compared to new TB patients. Comparable findings have been reported in Bangladesh, Azerbaijan, Tanzania, and Namibia, where treatment history was identified as the sole significant risk factor [15, 20, 23, 30]. This consistent association across diverse settings underscores the importance of completing and delivering high-quality first-line treatment to prevent the development of resistance. In addition to treatment history, a study from Thailand identified the age group 45–54 years and male sex as significant risk factors for RR-TB [18]. The absence of these associations in Nepal may reflect genuine epidemiological differences in the TB patient population or may indicate limited statistical power to detect such effects in subgroup analyses. Another study from Egypt found that comorbidity with bronchial asthma was the strongest predictor of RR-TB [31], a factor not assessed in the present study.

## Limitations

This survey has several limitations. First, it included only government facilities and excluded private providers. However, as most of the tuberculosis treatment is provided free of charge and the government providers cover most of the share for TB treatment and care services, the private sector’s impact is considered minimal. Additionally, 15.6% of samples were excluded from analysis due to contamination, lack of growth, or growth of non-tuberculous mycobacteria. Despite these limitations, the proportion of samples testing positive for MTBC in this survey was higher than that reported in Namibia (79.5%) [20], Mongolia (78%) [16], and Tanzania (78%) [23]. Furthermore, HIV status was missing for approximately half of the enrolled patients because interviews were conducted at DOTS initiation points before most patients had received their HIV test results, making retrospective data retrieval unfeasible. This limitation restricts the ability to fully characterize HIV as a potential risk factor for drug resistance and may reduce statistical power in subgroup analyses involving HIV status. Nevertheless, given Nepal's low national HIV prevalence (0.13%) and routine program data indicating that approximately 0.6% of TB patients are HIV co-infected, the likely impact of this data gap on overall resistance prevalence estimates is expected to be minimal [9, 32].

## Conclusions

The sixth national drug-resistant tuberculosis survey revealed that the prevalence of RR/MDR-TB among new patients is consistent with findings from the 2010/11 survey. This stability suggests transmission of resistant strains has persisted at a baseline level, while this may also reflect adherence to standardized treatment protocols; a meaningful reduction in the resistance burden remains unsubstantiated. Previous tuberculosis treatment was identified as the strongest risk factor for rifampicin resistance, whereas isoniazid resistance was the most prevalent overall. The high rate of fluoroquinolone resistance among new patients highlights the urgent need to expand drug-susceptibility testing, enhance antimicrobial stewardship, and implement stricter regulation of antibiotic use in both human and animal health sectors to prevent further emergence of DR-TB in Nepal.

## Supplementary Information

Supplementary Material 1.

Supplementary Material 2.

Supplementary Material 3.

## Data Availability

The datasets generated and/or analyzed during the current study are available from the corresponding author on reasonable request.

## Abbreviations

a*OR*: Adjusted odds ratio
*CI*: Confidence interval
DOTS: Directly observed treatment, short-course
DR-TB: Drug-resistant tuberculosis
DST: Drug susceptibility testing
EQA: External quality assurance
Hr-TB: Isoniazid mono-resistant tuberculosis
LJ: Lowenstein-Jensen
LPA: Line probe assay
MDR: Multidrug-resistant
MGIT: Mycobacteria growth indicator tube
MTBC: Mycobacterium tuberculosis complex
NTP: National tuberculosis program
*p*DST: Phenotypic drug susceptibility testing
PTB: Pulmonary tuberculosis
RR-TB: Rifampicin-resistant tuberculosis
*SD*: Standard deviation
SRL: Supranational reference laboratory
TB: Tuberculosis
XDR-TB: Extensively drug-resistant tuberculosis
Xpert MTB/RIF: Xpert *Mycobacterium tuberculosis*/Rifampicin assay
