# Supplementary material for: Drug-resistant tuberculosis in Nepal: the sixth national prevalence drug-resistant survey, 2024
Source: Infect Dis Poverty. 2026 Oct 13;15:109. doi: 10.1186/s40249-026-01503-z (PMC13660163; doi:10.1186/s40249-026-01503-z)
Supplement: Supplementary file 1 [file 40249_2026_1503_MOESM1_ESM.pdf]

Sample Size

The sample size for new registered cases was determined using the formula specified in WHO's Guidelines for Surveillance of Drug Resistance in TB *(World Health Organization, 2015)*:

$$n\ (SRS) = \frac{N \times z^2 \times p \times (1-p)}{d^2 \times (N-1) + z^2 \times p \times (1-p)}$$

Where:

n(SRS): Required sample size for simple random sampling

N = total number of new bacteriologically confirmed pulmonary TB cases registered in 2019 (12,626)[1] ;

z = z-value (from the standard normal distribution) that corresponds to the desired confidence level for a 95% confidence interval (1.96)

d = absolute precision (0.010)

p = expected proportion of RR/MDR-TB among new cases in 2019 (0.022)

To address the cluster-based design and the possible correlation between individuals within a cluster, a design effect of 2 was assumed, effectively doubling the sample size. Additionally, a 10% anticipated non-response rate was accounted for using a response rate adjustment factor of (R) of 0.9.

Total Sample size of New TB cases = [(SRS * DEFF) / r]

Substituting the values:

Total Sample Size= [(775.8 * 2)/0.9] = 1724

Thus, the final sample size for new registered bacteriologically confirmed pulmonary TB patients was determined to be 1,724.

All eligible previously treated TB patients consecutively enrolled until the required sample size for new patients was achieved within each cluster. Based on 2020 data, previously treated cases constituted approximately 11% of all bacteriologically confirmed pulmonary TB cases. This proportion indicated that around 190 previously treated patients were expected to be enrolled in

[1] Estimated number of new cases, if 89% of bacteriologically confirmed cases by either microscopy or Xpert MTB/RIF in the 2019 sampling frame (which reports new cases and relapses combined) are assumed to correspond to new cases. For mixed facilities offering both Xpert MTB/RIF and microscopy diagnostic services, a conservative caseload was taken for each centre which corresponded to the maximum number of cases diagnosed by either microscopy or Xpert MTB/ RIF (i.e. whichever was greater), due to the inability to ascertain the number of patients undergoing both tests (data not available). The resultant number was rounded down to the lower integer, for a conservative estimate of caseload at each health facility

the study. Therefore, overall, around 1914 patients (1724 plus 190) were estimated to be enrolled in the DRS.

Sampling Strategy and Sampling Frame

A stratified sampling strategy was employed to achieve the target of 1724 new cases, selecting 50 clusters (sites/health facilities) of varying sizes that were logistically feasible. The sampling frame comprised TB diagnostic centers, stratified by annual caseloads in 2020 into three strata: Stratum 1 (6–30 cases) with 146 sites, Stratum 2 (31–250 cases) with 73 sites, and Stratum 3 (>250 cases) with nine sites. The sample size was proportionally distributed across these strata, with 176 cases in Stratum 1, 934 in Stratum 2, and 614 in Stratum 3. Clusters were independently sampled using constant probability without replacement from each of the two smallest strata (Stratum 1: 21 clusters; Stratum 2: 20 clusters), while exhaustive sampling of all nine facilities was used in Stratum 3. A total of 50 clusters were selected, and the list of selected clusters is provided in Annex I. Patient enrollment was planned across all strata during fixed intake periods: 223 days (~7.4 months) for Stratum 1, 157 days (~5.2 months) for Stratum 2, and 52 days (~1.7 months) for Stratum 3. Collected samples were distributed between two central laboratories, NTRL/NTCC (from Koshi, Madesh, Bagmati, Gandaki, and Lumbini provinces) and GENETUP (from Karnali and Sudurpaschim). This sampling and processing strategy ensured representative data collection, operational feasibility, and adherence to quality standards.

Stratification of Cluster (Health Facility)

Stratum	Facility ID	Cluster ID	District	Diagnostic Centre	new name	Servi ces	2020 Caseload *
2020 Caseload: 6-30	HF_246	Cl_01	CHITAWAN	MANGALPUR HP_CHITAWAN		MC	6
2020 Caseload: 6-30	HF_716	Cl_02	DOTI	Shaileshwori Hospital_Doti		MC	6
2020 Caseload: 6-30	HF_124	Cl_03	DHANUSA	DEURI PARBAHA PHC_DHANUSA		MC	8
2020 Caseload: 6-30	HF_247	Cl_04	CHITAWAN	MEGHAULI HP_CHITAWAN		MC	8
2020 Caseload: 6-30	HF_339	Cl_05	MAKWANPUR	SHREEPUR CHHATIWAN PHC_MAKWANPUR	Bakiya Hospital	MC	8
2020 Caseload: 6-30	HF_355	Cl_06	RAMECHHAP	MANTHALI PHC _RAMECHHAP		MC	8
2020 Caseload: 6-30	HF_620	Cl_07	JAJARKOT	DISTRICT HOSPITAL_ JAJARKOT		MC	8
2020 Caseload: 6-30	HF_724	Cl_08	KAILALI	DARAKH HP_KAILALI		MC	8
2020 Caseload: 6-30	HF_762	Cl_09	KANCHANPUR	CHANDANI HP_KANCHANPUR		MC	8
2020 Caseload: 6-30	HF_627	Cl_10	MUGU	DISTRICT HOSPITAL_ MUGU		MC	9
2020 Caseload: 6-30	HF_453	Cl_11	TANAHU	BHIMAD PHC_TANAHU		MC	11
2020 Caseload: 6-30	HF_211	Cl_12	SIRAHA	BHAGWANPUR PHC_SIRAHA		MC	12
2020 Caseload: 6-30	HF_751	Cl_13	KAILALI	SADEPANI HP_KAILALI		MC	13
2020 Caseload: 6-30	HF_651	Cl_14	ACHHAM	BINAYAK HP_ACHHAM		MC	16
2020 Caseload: 6-30	HF_720	Cl_15	KAILALI	BHAJANEE PHC_KAILALI		MC	17
2020 Caseload: 6-30	HF_248	Cl_16	CHITAWAN	NATIONAL ANTI TUBERCLOSIS ASSOCIATION_CHITAWAN		MC	19
2020 Caseload: 6-30	HF_294	Cl_17	KATHMANDU	NEPAL POLICE HOSPITAL_KATHMANDU		MC, GX	20
2020 Caseload: 6-30	HF_684	Cl_18	BAJHANG	DISTRICT HOSPITAL_ BAJHANG		MC	23
2020 Caseload: 6-30	HF_263	Cl_19	DHADING	SALYANTAR PHC_DHADING		MC	25
2020 Caseload: 6-30	HF_763	Cl_20	KANCHANPUR	DAIJEE HP_KANCHANPUR		MC	26
2020 Caseload: 6-30	HF_574	Cl_21	ROLPA	ROLPA HOSPITAL REUGHA		MC	27

2020 Caseload: 31 - 250	HF_353	Cl_22	SINDHULI	SIDDESHOR HP_SINDHULI	MC	32
2020 Caseload: 31 - 250	HF_103	Cl_23	MORANG	NOBEL MEDICAL COLLAGE_MORANG	GX	34
2020 Caseload: 31 - 250	HF_204	Cl_24	MAHOTTARI	RAMGOPALPUR HP _MAHOTTARI	MC	35
2020 Caseload: 31 - 250	HF_118	Cl_25	SARLAHI	BARAHATHAWA PHC _SARLAHI	MC	37
2020 Caseload: 31 - 250	HF_394	Cl_26	NAWALPARASI	CHORMARA PHC_NAWALPARASI	MC	37
2020 Caseload: 31 - 250	HF_643	Cl_27	SALYAN	DISTRICT HOSPITAL_SALYAN	GX	52
2020 Caseload: 31 - 250	HF_185	Cl_28	SARLAHI	MALANGAWA DISTRICT HOSPITAL_ SARLAHI	MC, GX	55
2020 Caseload: 31 - 250	HF_775	Cl_29	KAILALI	TIKAPUR HOSPITAL_ KAILALI	MC, GX	64
2020 Caseload: 31 - 250	HF_248	Cl_30	BHAKTAPUR	CHANGUNARAYAN PHC _BHAKTAPUR	MC	69
2020 Caseload: 31 - 250	HF_140	Cl_31	DHANUSA	DHALKEBAR HP_DHANUSA	MC, GX	92
2020 Caseload: 31 - 250	HF_242	Cl_32	KATHMANDU	Civil Hospital	GX	92
2020 Caseload: 31 - 250	HF_323	Cl_33	MAKAWANPUR	NAKAULI URBAN HEALTH CENTRE_MAKAWANPUR	MC	98
2020 Caseload: 31 - 250	HF_534	Cl_34	PALPA	LUMBINI MEDICAL COLLEGE_PALPA	MC, GX	99
2020 Caseload: 31 - 250	HF_567	Cl_35	KAPILBASTU	PIPARA HOSPITAL_KAPILBASTU	MC, GX	155
2020 Caseload: 31 - 250	HF_478	Cl_36	RUPANDEHI	BHIM HOSPITAL_ RUPANDEHI	MC, GX	157
2020 Caseload: 31 - 250	HF_568	Cl_37	NAWALPARASI	PRITHIV CHANDRA HOSPITAL_ NAWALPARASI	MC, GX	188
2020 Caseload: 31 - 250	HF_133	Cl_38	RAUTAHAT	CHANDRANIGAHAPUR HOSPTIAL_ RAUTAHAT	MC, GX	206
2020 Caseload: 31 - 250	HF_110	Cl_39	BARA	DISTRICT HOSPITAL_BARA	MC, GX	215

2020 Caseload: 31 - 250	HF_17	Cl_40	SUNSARI	BPKIHS_ DHARAN_SUNSARI		MC, GX	218
2020 Caseload: 31 - 250	HF_243	Cl_41	KATHMANDU	HERD laboratory		GX	234
2020 Caseload: >250	HF_67	Cl_42	MORANG	MANGALBARE Hospital MORANG	Madan Bhandari Trauma Center, UrlaBari	MC, GX	265
2020 Caseload: >250	HF_70	Cl_43	MORANG	NEPAL ANTI TUBERCLOSIS ASSOCIATION (NATA)_MORANG		MC, GX	362
2020 Caseload: >250	HF_190	Cl_44	PARSA	NARAYANI DOTS CLINIC BIRGUNG_208		MC, GX	619
2020 Caseload: >250	HF_231	Cl_45	BHAKTAPUR	NATIONAL TUBERCULOSIS CONTROL CENTER		MC, GX	753
2020 Caseload: >250	HF_284	Cl_46	KATHMANDU	KALIMATI CHEST HOSPITAL (NATA)_KATHMANDU		MC, GX	679
2020 Caseload: >250	HF_407	Cl_47	KASKI	DPHO DOTS CENTRE/WRTC Kaski		MC, GX	309
2020 Caseload: >250	HF_569	Cl_48	RUPENDEHI	PROVINCIAL HOSPITAL LUMBINI RUPANDEHI		MC, GX	406
2020 Caseload: >250	HF_600	Cl_49	BANKE	TB-Nepal_ Banke		MC, GX	621
2020 Caseload: >250	HF_762	Cl_50	KAILALI	SETI ZONAL HOSPITAL_ KAILALI		MC, GX	316
