# Supplementary material for: Drug-resistant tuberculosis in Nepal: the sixth national prevalence drug-resistant survey, 2024
Source: Infect Dis Poverty. 2026 Oct 13;15:109. doi: 10.1186/s40249-026-01503-z (PMC13660163; doi:10.1186/s40249-026-01503-z)
Supplement: Supplementary file 2 [file 40249_2026_1503_MOESM2_ESM.pdf]

Clinical Information Form

नेपाल सरकार
स्वास्थ्य तथा जनसंख्या मन्त्रालय
राष्ट्रिय क्षयरोग नियन्त्रण केन्द्र
क्षयरोगको औषधी प्रतिरोधी अवस्थाको राष्ट्रिय सर्वेक्षण
(National Anti-Tuberculosis Drug Resistance Survey)

क्लिनिकल सूचना फारम

(Clinical Information Form)

सि.नं	प्रश्नहरु	सम्भावित उत्तर तथा कोड	निर्देशन
**खण्ड १. उत्तरदाता तथा स्वास्थ्य संस्थाको विवरण**			
१.१	DRS बिरामी संकेत नं<br>DRS Patient ID (Scan Barcode)	*(Paste barcode of DRS Patient ID here)*<br>–	
१.२	Date of Interview:<br>dd/mm/yyyy	*(गते/महिना/साल)*<br>dd/mm/yyyy<br>_____/ _____/ ________	
१.३	निदान केन्द्रको नाम<br>Cluster Name	___________________________	
१.४	प्रदेश<br>Province:	१ प्रदेश नं १ (Province 1)	
		२ मधेश प्रदेश(Madhesh Province)	
		३ बागमति प्रदेश(Bagmati Province)	
		४ गण्डकी प्रदेश (Gandaki Province)	
		५ लुम्बिनी प्रदेश (Lumbini Province)	
		६ कर्णाली प्रदेश(Karnali Province)	
		७ सूदुरपश्चिम प्रदेश(Sudurpaschim Province)	
१.५	जिल्ला District:	_________________	
१.६	पालिका Palika:	_________________	
१.७	वार्ड नं Ward no:	_____	
१.८	उपलब्ध क्षयरोग परिक्षण<br>*(एक भन्दा बढि उत्तर आउन सक्छ)*<br>Available TB Diagnostic Tests	१ स्मियर माइक्रोस्कोपि Smear Microscopy	
		२ जिन एक्सपर्ट GeneXpert	
		३ कल्चर Culture	
		४ LPA	

सि.नं	प्रश्नहरु	सम्भावित उत्तर तथा कोड		निर्देशन
**खण्ड २: खकारका नमुना सम्बन्धी विवरण SECTION 2: DRS SPUTUM SAMPLE**				
२.१	पहिलो खकारको नमुनाको संकेत नं<br>Sample A DRS ID (Scan Barcode)	*(Paste first sample's barcode here)*		
२.२	पहिलो खकारको नमुना संकलन मिति<br>Sample A Collection Date		*(गते/महिना/सालं)*<br>dd/mm/yyyy<br>_____/ _____/ ________	
२.३	दोस्रो खकारको नमुनाको संकेत नं<br>Sample B DRS ID (Scan Barcode)	*(Paste second sample's barcode here)*		
२.४	दोस्रो खकारको नमुना संकलन मिति<br>Sample B Collection Date		*(गते/महिना/सालं)*<br>dd/mm/yyyy<br>_____/ _____/ ________	
**SECTION 3: PERIPHERAL SPUTUM SMEAR MICROSCOPY AND XPERT MTB/RIF TEST RESULTS**				
३.१	तपाईंको स्मियर माइक्रोस्कोपिबाट खकार परिक्षण भएको छ ? Diagnosed through smear microscopy?	१	छ (Yes)	*छैन भने प्र.नं. ३.५ मा जाने*
		२	छैन (No)	
३.२	स्मियर माइक्रोस्कोपिबाट परिक्षण भएको निदान केन्द्रको नाम Name of microscopic center		____________________	
३.३	स्मियर माइक्रोस्कोपिबाट परिक्षण भएको मिति<br>Date of smear microscopy		*गते/महिना/साल)*<br>dd/mm/yyyy<br>_____/ _____/ ________	
३.४	स्मियर माइक्रोस्कोपिको नतिजा<br>Result of smear microscopy	१	Negative	
		२	Scanty	
		३	1+	
		४	2+	
		५	3+	
३.५	तपाईंको जिन एक्सपर्टबाट खकार परिक्षण भएको छ ? Diagnosed through GeneXpert test?	१	छ (Yes)	***छैन भने खण्ड ४. मा जाने***
		२	छैन (No)	
३.६	जिन एक्सपर्टबाट परिक्षण भएको निदान केन्द्रको नाम Name of GeneXpert center		____________________	
३.७	जिन एक्सपर्ट परिक्षण भएको मिति<br>Date of Xpert MTB/RIF Test		*गते/महिना/साल)*<br>dd/mm/yyyy<br>_____/ _____/ ________	
३.८	स्थानीय स्वास्थ्य संस्थामा गरिएको जिन एक्सपर्ट परिक्षणको नतिजा :<br>Result of Xpert MTB/RIF Test	१	*MTB-Not Detected*	
		२	*MTB Detected Rif Sensitive*	
		३	*MTB Detected Rif Resistant*	
		४	*MTB Detected Rif indeterminate*	

सि.नं	प्रश्नहरु	सम्भावित उत्तर तथा कोड		निर्देशन
		५	*Invalid*	
		६	*No result*	
		७	*Error*	
**खण्ड ४: बिरामीको विवरण** t (**SECTION 4: PATIENT DETAILS)**				
४.१	बिरामीको पूरा नाम र थर (Patient's Full Name)		______________________	
४.२	जात/जातीयताः (Caste/Ethnicity)______		कोड (Code) ____	
४.३	लिङ्ग (Sex)	१	महिला (Female)	
		२	पुरुष (Male)	
		९६	अन्य (Others)	
४.४	उमेर (Age)		(पूरा भएको वर्ष) (completed years): _______	
		९८	थाहा छैन (Don't Know)	
४.५	जन्म मिति (Date of birth)		*गते/महिना/साल)* (dd/mm/yyyy) _____/ _____/ ________	
		९८	थाहा छैन (Don't Know)	
४.६	यदि, बिरामी क्षयरोग उपचारका लागि दर्ता भइसकेको भए, क्षयरोग दर्ता नं (If already registered, TB Register Number:)		______________________	
४.७	मोबाइल नं (Patient's mobile number)		______________________	
४.८	अन्य सम्पर्क नं (Other contact Nnmber)		______________________	
४.९	हाल अक्सर बसोबार गर्ने ठेगाना (६ महिना बढिबसोबास गरेको) (Current Residence ≥6 months stay)			
४.९.१	प्रदेश (Province)	१	प्रदेश नं १ (Province 1)	
		२	मधेश प्रदेश(Madhesh Province)	
		३	बागमति प्रदेश(Bagmati Province)	
		४	गण्डकी प्रदेश (Gandaki Province)	
		५	लुम्बिनी प्रदेश (Lumbini Province)	
		६	कर्णाली प्रदेश(Karnali Province)	
		७	सूदुरपश्चिम प्रदेश(Sudurpaschim Province)	

सि.नं	प्रश्नहरु	सम्भावित उत्तर तथा कोड		निर्देशन
४.९.२	जिल्ला (District)___________________		कोड (Code) ____	
४.९.३	पलिका (Palika) ____________________		कोड (Code)____	
४.९.४	वार्ड नं (Ward no.)		_____	
४.१०	जन्मेको देश (Country of Origin)<br>*(नेपाल भए ४.११ म जाने, अन्य देश भए ४.१५ मा जाने) जन्मस्थानको आधारमा लेख्ने*		______________________	
४.११	प्रदेश (Province)	१	प्रदेश नं १ (Province 1)	
		२	मधेश प्रदेश(Madhesh Province)	
		३	बागमति प्रदेश(Bagmati Province)	
		४	गण्डकी प्रदेश (Gandaki Province)	
		५	लुम्बिनी प्रदेश (Lumbini Province)	
		६	कर्णाली प्रदेश(Karnali Province)	
		७	सूदुरपश्चिम प्रदेश(Sudurpaschim Province)	
४.१२	जिल्ला (District)___________________		कोड (Code) ____	
४.१३	पलिका (Palika)___________________		कोड (Code)____	
४.१४	वार्ड नं (Ward no.)		______	
४.१५	एच. आइ. भि. परिक्षण भएको छ? (HIV test done?)	१	छ (Yes)	***छैन वा थाहा छैन भने खण्ड ५ मा जाने***
		२	छैन (No)	
		९८	थाहा छैन (Don't know)	
४.१६	एच. आइ. भि. परिक्षण भएको मिति (Date of HIV test)		*गते/महिना/साल)*<br>(dd/mm/yyyy)<br>_____/ _____/ ________	
४.१७	एच. आइ. भि. परिक्षणको नतिजा: (HIV Status)	१	Negative	
		२	Positive	
		३	Unknown	
**खण्ड ५: बिरामीको रोगको इतिहास (SECTION 5: HISTORY GIVEN BY THE PATIENT)**				
५.१	विगतमा तपाईंको क्षयरोगको उपचार १ महिना भन्दा बढि समयलाई भएको थियो ?<br>Previously treated for TB?	१	छ (Yes)	
		२	छैन (No)	
		९८	थाहा छैन (Don't know)	

यदि प्रश्न नं ५.१ को उत्तर "थिएन" वा "थाहा छैन" आएमा बिरामीको क्षयरोगको उपचारको इतिहासबारे थप निश्चित गर्न प्रश्न नं ५.२ मा जानुहोस् र यदि उत्तर "थियो" आएमा बिरामीको उपचार कार्ड वा उपचार खुलाउने अन्य पत्र/प्रतिवेदनको आधारमा खण्ड ६ पूरा गर्नुहोस् ।

If 'No' to Question 5.1, go to Question 18. If 'Yes' to Question Section 5.1, go to section 6.

सि.नं	प्रश्नहरु	सम्भावित उत्तर तथा कोड		निर्देशन
५.२	तपाईं कति लामो समयदेखि बिरामी हुनु हुन्छ ?<br>For how long you have been sick?		________ दिन	
५.३	तपाईंलाई यस अघि क्षयरोग वा फोक्सो सम्बन्धि रोगको लक्षणहरु देखा परेका थिए? (जस्तैः खोकी लाग्ने, छाती दुख्ने, खकारमा रगत आउने आदि)<br>*(अहिले क्षयरोग देखा पर्नु भन्दा अगाडिको घटनाको आधारमा पूरा गर्नुहोस् ।)*<br>Had similar TB related symptoms prior to this episode?	१	छ (Yes)	***थिएन वा थाहा छैन आएमा प्र.न.५.५ मा जाने***
		२	छैन (No)	
		९८	थाहा छैन (Don't know)	
५.४	पहिले कस्ता लक्षणहरु देखा परेका थिए ?<br>*(एक भन्दा बढि उत्तर आउन सक्ने)*<br>Types of symptoms prior to this episode?	१	खोकी लाग्ने (Cough)	
		२	खकारमा रगत आउने	
		३	छाती दुख्ने (Chest pain)	
		४	तौल घट्ने (Weight lost)	
		९६	अन्य (उल्लेख गर्नुहोस्)<br>Other (specify)<br>________________________	
५.५	यस अघि क्षयरोग निदानका लागि खकार परिक्षण गर्नु भएको थियो ?Had sputum examinations prior to this episode?	१	छ (Yes)	
		२	छैन (No)	
		९८	थाहा छैन (Don't know)	
५.६	यस अघि क्षयरोगको औषधि एक महिना भन्दा बढिको लागि सेवन गर्नु भएको थियो ?<br>Took TB drugs for more than one month?	१	छ (Yes)	***थिएन वा थाहा छैन आएमा प्र.नं.५.८ मा जाने***
		२	छैन (No)	
		९८	थाहा छैन (Don't know)	
५.७	यदि "छ" भने, औषधिहरुको नाम उल्लेख गरिदिनुहोला । If yes, what were the drugs?		________________________	
		९८	थाहा छैन (Don't know)	
५.८	यस अघि क्षयरोगको उपचारको लागि एक महिना भन्दा बढि सुई लगाउनु भएको थियो ? Had injections for TB for more than one month?	१	छ (Yes)	
		२	छैन (No)	
		९८	थाहा छैन (Don't know)	
५.९	माथि उल्लेखित प्रश्नहरु सोधेपछि के बिरामीले आफ्नो बिगतको क्षयरोगको उपचार बारेमा सम्झिनु भयो ? Patient remembered previous treatment history for TB after probing?	१	सम्झिनु भयो (Remembered)	
		२	सम्झिनु भएन (Did not Remember)	
**खण्ड ६: चिकित्सा विवरण (SECTION 6: MEDICAL RECORDS)**				
६.१		१	छ (Yes)	***छैन वा थाहा छैन***
		२	छैन (No)	

सि.नं	प्रश्नहरु	सम्भावित उत्तर तथा कोड		निर्देशन
	कागज पत्र हेर्दा बिरामी यस अघि क्षयरोगको उपचारको लागि दर्ता भइ १ महिना भन्दा बढी क्षयरोगको औषधी लिएको पुष्टि भएको छ ? After extensive checking through the medical files and other documents available in the health centre, have you discovered that the patient has been registered for tuberculosis treatment for more than a month before?	९८	थाहा छैन (Don't know)	*आएमा प्र.नं.६.५ मा जाने*
६.२	यदि यस अघि १ महिना भन्दा बढी क्षयरोगको औषधी लिएको भए सो औषधी कहिले लिएका थिए ? How long ago was the patient treated for TB for more than 1 month?		____ वर्ष अगाडि Years: ___<br>____ महिना अगाडिMonths: _<br>____ हप्ता अगाडिWeeks: ____<br>____ दिन अगाडिDays: ____	
६.३	उपचारमा दर्ता मिति<br>Previous TB registration date		*गते/महिना/साल)*<br>(dd/mm/yyyy)<br>_____/ _____/ ________	
६.४	यस अघिको क्षयरोग दर्ता नं :<br>Previous TB registration number		________________	
**यदि प्रश्न नं ५.१, ५.६, ५.८, ६.१ को उत्तर "छ" वा "थियो" भएमा प्र.नं. ६.५ मा "छ" उत्तरमा O चिन्ह लगाउनुहोस् ।**				
६.५	बिरामी यस अघि क्षयरोगको उपचारमा १ महिना भन्दा रहेको छ ? Patient has been previously treated for TB for more than a month?	१	छ (Yes)	*थिएन वा थाहा छैन आएमा खण्ड ७ मा जानेे*
		२	छैन (No)	
		९८	थाहा छैन (Don't know)	
६.६	विगतको क्षयरोगको उपचारको नतिजा के थियो ? *If 'Yes' to Question 6.5,* what was the outcome of previous treatment?	१	निको भएको / उपचार पूर्ण गरिएको Cured/treatment completed	
		२	नयाँ पहिलो चरणको औषधि उपचार मात्र असफल भएको Failed new patient regimen of first-line drugs only	
		३	पहिलो चरणको पुनः गरिएको औषधि उपचार मात्र असफल भएको Failed retreatment regimen of first-line drugs only	
		४	दोश्रो चरणको औषधि उपचार असफल भएको Failed regimen which included second-line drugs	

सि.नं	प्रश्नहरु		सम्भावित उत्तर तथा कोड	निर्देशन
		५	उपचार पुर्ण नभई बिरामी हराएको Lost to follow-up	
		९६	अन्य Other	
		९८	थाहा नभएको Unknown	
**खण्ड ७: जोखिम कारकहरुको विवरण**				
७.१	तपाईं हाल धुम्रपान सेवन गर्नुहुन्छ? (Are you a current smoker?)	१	छ (Yes)	
		२	छैन (No)	
७.२	तपाईंलाई मधुमेह रोग निदान भएको छ? (Have you been diagnosed with diabetes mellitus?)	१	छ (Yes)	
		२	छैन (No)	
७.३	तपाईंलाई हाल अन्य कुनै दीर्घरोग निदान भएको छ?(जस्तैः उच्च रक्तचाप, क्यान्सर, मुटुरोग, दम, मानसिक रोग आदि) (Do you have other chronic illness?)	१	छ (Yes)	*छैन आएमा प्र.नं ७.५ मा जाने*
		२	छैन (No)	
७.४	तपाईंलाई के–के दीर्घरोग (हरु) निदान भएको छ? (Is yes, what chronic illnesses do you have?)		______________________	
७.५	तपाईंको मुख्य पेशा के हो? (Main occupation?)	१	दैनिक ज्याला मज्दूरी (Daily wage worker)	
		२	सरकारी कर्मचारी (Government service)	
		३	निजी संस्थाको कर्मचारी (Private service)	
		४	स्वरोजगारी (Self employed)	
		५	बैदेशिक रोजगारी (Foreign employment)	
		६	बेरोजगार(Unemployment)	
		९६	अन्य (Others)......................	
**यदि प्र.नं.७.५ को उत्तर "वैदेशिक रोजगार" भएमा प्र.नं ७.६ मा जाने अन्यथा सिधै प्र.नं ८ मा जानुहोस् ।**				
७.६	हाल रोजगारी गरिरहेको देशको नाम (If Foreign employment, recent place of employment?)		__________________	
७.७	तपाईं उक्त देशमा कुन पेशा गरिरहनु भएको छ? (What is your occupation in foreign country?)		__________________	
७.८	तपाईं कति समयदेखि नेपाल बाहिर रोजगारी गर्दै हुनुहुन्छ? (How long have you been working outside Nepal?)		____ वर्ष (Years)<br>____ महिना (Months)<br>____ हप्ता (Weeks)<br>____ दिन (Days)	

सि.नं	प्रश्नहरु	सम्भावित उत्तर तथा कोड		निर्देशन
७.९	तपाईं वर्षको कति पटक नेपाल आउने गर्नुहुन्छ? (How often do you visit Nepal?)		____ पटक (Frequency)	
७.१०	शैक्षिक स्तर (Education)		पूरा गरको कक्षा वा तह (Completed grade or level) __________	
		९४	अनौपचारिक शिक्षा (Informal education)	
		९५	निरक्षर/लेखपढ गर्न नसक्ने (Illiterate/can't read and write)	
८	अन्तर्वार्ता लिनेको नाम र सहि<br><br>Interviewer's Name:			
९	फोकल व्यक्तिको नाम र सहि<br>DOTS Focal Person's Name			

तपाईंको अमूल्य समय र जानकारीको लागि धन्यवाद।

(Thank you for your valuable information and time)

TB Diagnostic Centre Shipment Form

Government of Nepal
Ministry of Health and Population
National Tuberculosis Control Center
National Anti-TB Drugs Resistance Survey

DRS 2.1. TB Diagnostic Centre Shipment Form

Name of Cluster: ________________________________

Cluster ID: _________

DRS Patient ID	Sample A collected (Date)	Sample B collected (Date)	Forms checked and added to shipment	Date of Shipment *(DD/MM/YYYY)*	Person Packing	Person Collecting for Shipment and Phone Number
(Paste Barcode here)	(___/___/____)	(___/___/____)	☐ CIF ☐Consent ☐Shipment			( )
(Paste Barcode here)	(___/___/____)	(___/___/____)	☐ CIF ☐Consent ☐Shipment			( )
(Paste Barcode here)	(___/___/____)	(___/___/____)	☐ CIF ☐Consent ☐Shipment			( )
(Paste Barcode here)	(___/___/____)	(___/___/____)	☐ CIF ☐Consent ☐Shipment			( )

Central Laboratory Register

Age	Sex	Name of Referral Cluster Site	TB Registration Number	New (N) /Previously Treated (P)	Sample collection date *(DD/MM/YYYY)* and Time		Sample Received Date *(DD/MM/YYYY)* and Time	Temperature (°C) of received samples	Sample quality		Microscopy Result		Gene Xpert		LJ culture result					MGIT DST Result																Line Probe Assay (LPA) result																
					Sample A	Sample B			Sample A	Sample B	Sample A	Sample B	Peripheral Lab Result	Central Lab Result	Inoculation Date *(DD/MM/YYYY)*	Sample A	Sample B	MPT_64	Final Result	Primary inoculum for DST	Inoculation Date *(DD/MM/*	Result Date *(DD/MM/YYYY)*	H 0.1 µg/ml	R 0.5 µg/ml	E 5.0 µg/ml	Z 100 µg/ml	Lfx 1.0 µg/ml	Mfx 0.25 µg/ml	Mfx 1.0 µg/ml	Amk 1.0 µg/ml	Cfz 1.0 µg/ml	Lzd 1.0 µg/ml	Dlm 0.06 µg/ml	Bdq 1.0 µg/ml	Other drugs ------	Date of Processing *(DD/MM/YYYY)*	Direct or Culture	MTB Complex	Hybridization result and interpretation														
																																							rpoB	R	*katG Ba*	*inhA Ba*	H	*gyrA Ba*	*gyrB Ba*	FLQ	*rrs Ba*	*eis Ba*	AMK	KM	CM	ETI
									1.S 2.M 3.B 4.O	1.S 2.M 3.B 4.O	1.Neg 2. Scanty 3.1+ 4.2+ 5.3+	1.Neg 2. Scanty 3.1+ 4.2+ 5.3+	1.MTB ND 2.Rif Sen. 3.Rif Res. 4.Rif Ind.	1.MTB ND 2.Rif Sen. 3.Rif Res. 4.Rif Ind.					1.MTBC 2. NTM 3. Neg. 4. Cont.	1.Solid 2. Liquid			1.R 2.S 3.C 4.NA	1.R 2.S 3.C 4.NA	1.R 2.S 3.C 4.NA	1.R 2.S 3.C 4.NA	1.R 2.S 3.C 4.NA	1.R 2.S 3.C 4.NA	1.R 2.S 3.C 4.NA	1.R 2.S 3.C 4.NA	1.R 2.S 3.C 4.NA	1.R 2.S 3.C 4.NA	1.R 2.S 3.C 4.NA	1.R 2.S 3.C 4.NA	1.R 2.S 3.C 4.NA		1.Direct Specimen 2. Culture Positive	1.Detected 2.Not Detected		1.R 2.S 3.I			1.R 2.S 3.I			1.R 2.S 3.I			1.R 2.S 3.I	1.R 2.S 3.I	1.R 2.S 3.I	1.R 2.S 3.I
									1.S 2.M 3.B 4.O	1.S 2.M 3.B 4.O	1.Neg 2. Scanty 3.1+ 4.2+ 5.3+	1.Neg 2. Scanty 3.1+ 4.2+ 5.3+	1.MTB ND 2.Rif Sen. 3.Rif Res. 4.Rif Ind.	1.MTB ND 2.Rif Sen. 3.Rif Res. 4.Rif Ind.					1.MTBC 2. NTM 3. Neg. 4. Cont.	1.Solid 2. Liquid			1.R 2.S 3.C 4.NA	1.R 2.S 3.C 4.NA	1.R 2.S 3.C 4.NA	1.R 2.S 3.C 4.NA	1.R 2.S 3.C 4.NA	1.R 2.S 3.C 4.NA	1.R 2.S 3.C 4.NA	1.R 2.S 3.C 4.NA	1.R 2.S 3.C 4.NA	1.R 2.S 3.C 4.NA	1.R 2.S 3.C 4.NA	1.R 2.S 3.C 4.NA	1.R 2.S 3.C 4.NA		1.Direct Specimen 2. Culture Positive	1.Detected 2.Not Detected		1.R 2.S 3.I			1.R 2.S 3.I			1.R 2.S 3.I			1.R 2.S 3.I	1.R 2.S 3.I	1.R 2.S 3.I	1.R 2.S 3.I
									1.S 2.M 3.B 4.O	1.S 2.M 3.B 4.O	1.Neg 2. Scanty 3.1+ 4.2+ 5.3+	1.Neg 2. Scanty 3.1+ 4.2+ 5.3+	1.MTB ND 2.Rif Sen. 3.Rif Res. 4.Rif Ind.	1.MTB ND 2.Rif Sen. 3.Rif Res. 4.Rif Ind.					1.MTBC 2. NTM 3. Neg. 4. Cont.	1.Solid 2. Liquid			1.R 2.S 3.C 4.NA	1.R 2.S 3.C 4.NA	1.R 2.S 3.C 4.NA	1.R 2.S 3.C 4.NA	1.R 2.S 3.C 4.NA	1.R 2.S 3.C 4.NA	1.R 2.S 3.C 4.NA	1.R 2.S 3.C 4.NA	1.R 2.S 3.C 4.NA	1.R 2.S 3.C 4.NA	1.R 2.S 3.C 4.NA	1.R 2.S 3.C 4.NA	1.R 2.S 3.C 4.NA		1.Direct Specimen 2. Culture Positive	1.Detected 2.Not Detected		1.R 2.S 3.I			1.R 2.S 3.I			1.R 2.S 3.I			1.R 2.S 3.I	1.R 2.S 3.I	1.R 2.S 3.I	1.R 2.S 3.I
									1.S 2.M 3.B 4.O	1.S 2.M 3.B 4.O	1.Neg 2. Scanty 3.1+ 4.2+ 5.3+	1.Neg 2. Scanty 3.1+ 4.2+ 5.3+	1.MTB ND 2.Rif Sen. 3.Rif Res. 4.Rif Ind.	1.MTB ND 2.Rif Sen. 3.Rif Res. 4.Rif Ind.					1.MTBC 2. NTM 3. Neg. 4. Cont.	1.Solid 2. Liquid			1.R 2.S 3.C 4.NA	1.R 2.S 3.C 4.NA	1.R 2.S 3.C 4.NA	1.R 2.S 3.C 4.NA	1.R 2.S 3.C 4.NA	1.R 2.S 3.C 4.NA	1.R 2.S 3.C 4.NA	1.R 2.S 3.C 4.NA	1.R 2.S 3.C 4.NA	1.R 2.S 3.C 4.NA	1.R 2.S 3.C 4.NA	1.R 2.S 3.C 4.NA	1.R 2.S 3.C 4.NA		1.Direct Specimen 2. Culture Positive	1.Detected 2.Not Detected		1.R 2.S 3.I			1.R 2.S 3.I			1.R 2.S 3.I			1.R 2.S 3.I	1.R 2.S 3.I	1.R 2.S 3.I	1.R 2.S 3.I
									1.S 2.M 3.B 4.O	1.S 2.M 3.B 4.O	1.Neg 2. Scanty 3.1+ 4.2+ 5.3+	1.Neg 2. Scanty 3.1+ 4.2+ 5.3+	1.MTB ND 2.Rif Sen. 3.Rif Res. 4.Rif Ind.	1.MTB ND 2.Rif Sen. 3.Rif Res. 4.Rif Ind.					1.MTBC 2. NTM 3. Neg. 4. Cont.	1.Solid 2. Liquid			1.R 2.S 3.C 4.NA	1.R 2.S 3.C 4.NA	1.R 2.S 3.C 4.NA	1.R 2.S 3.C 4.NA	1.R 2.S 3.C 4.NA	1.R 2.S 3.C 4.NA	1.R 2.S 3.C 4.NA	1.R 2.S 3.C 4.NA	1.R 2.S 3.C 4.NA	1.R 2.S 3.C 4.NA	1.R 2.S 3.C 4.NA	1.R 2.S 3.C 4.NA	1.R 2.S 3.C 4.NA		1.Direct Specimen 2. Culture Positive	1.Detected 2.Not Detected		1.R 2.S 3.I			1.R 2.S 3.I			1.R 2.S 3.I			1.R 2.S 3.I	1.R 2.S 3.I	1.R 2.S 3.I	1.R 2.S 3.I
									1.S 2.M 3.B 4.O	1.S 2.M 3.B 4.O	1.Neg 2. Scanty 3.1+ 4.2+ 5.3+	1.Neg 2. Scanty 3.1+ 4.2+ 5.3+	1.MTB ND 2.Rif Sen. 3.Rif Res. 4.Rif Ind.	1.MTB ND 2.Rif Sen. 3.Rif Res. 4.Rif Ind.					1.MTBC 2. NTM 3. Neg. 4. Cont.	1.Solid 2. Liquid			1.R 2.S 3.C 4.NA	1.R 2.S 3.C 4.NA	1.R 2.S 3.C 4.NA	1.R 2.S 3.C 4.NA	1.R 2.S 3.C 4.NA	1.R 2.S 3.C 4.NA	1.R 2.S 3.C 4.NA	1.R 2.S 3.C 4.NA	1.R 2.S 3.C 4.NA	1.R 2.S 3.C 4.NA	1.R 2.S 3.C 4.NA	1.R 2.S 3.C 4.NA	1.R 2.S 3.C 4.NA		1.Direct Specimen 2. Culture Positive	1.Detected 2.Not Detected		1.R 2.S 3.I			1.R 2.S 3.I			1.R 2.S 3.I			1.R 2.S 3.I	1.R 2.S 3.I	1.R 2.S 3.I	1.R 2.S 3.I
									1.S 2.M 3.B 4.O	1.S 2.M 3.B 4.O	1.Neg 2. Scanty 3.1+ 4.2+ 5.3+	1.Neg 2. Scanty 3.1+ 4.2+ 5.3+	1.MTB ND 2.Rif Sen. 3.Rif Res. 4.Rif Ind.	1.MTB ND 2.Rif Sen. 3.Rif Res. 4.Rif Ind.					1.MTBC 2. NTM 3. Neg. 4. Cont.	1.Solid 2. Liquid			1.R 2.S 3.C 4.NA	1.R 2.S 3.C 4.NA	1.R 2.S 3.C 4.NA	1.R 2.S 3.C 4.NA	1.R 2.S 3.C 4.NA	1.R 2.S 3.C 4.NA	1.R 2.S 3.C 4.NA	1.R 2.S 3.C 4.NA	1.R 2.S 3.C 4.NA	1.R 2.S 3.C 4.NA	1.R 2.S 3.C 4.NA	1.R 2.S 3.C 4.NA	1.R 2.S 3.C 4.NA		1.Direct Specimen 2. Culture Positive	1.Detected 2.Not Detected		1.R 2.S 3.I			1.R 2.S 3.I			1.R 2.S 3.I			1.R 2.S 3.I	1.R 2.S 3.I	1.R 2.S 3.I	1.R 2.S 3.I
									1.S 2.M 3.B 4.O	1.S 2.M 3.B 4.O	1.Neg 2. Scanty 3.1+ 4.2+ 5.3+	1.Neg 2. Scanty 3.1+ 4.2+ 5.3+	1.MTB ND 2.Rif Sen. 3.Rif Res. 4.Rif Ind.	1.MTB ND 2.Rif Sen. 3.Rif Res. 4.Rif Ind.					1.MTBC 2. NTM 3. Neg. 4. Cont.	1.Solid 2. Liquid			1.R 2.S 3.C 4.NA	1.R 2.S 3.C 4.NA	1.R 2.S 3.C 4.NA	1.R 2.S 3.C 4.NA	1.R 2.S 3.C 4.NA	1.R 2.S 3.C 4.NA	1.R 2.S 3.C 4.NA	1.R 2.S 3.C 4.NA	1.R 2.S 3.C 4.NA	1.R 2.S 3.C 4.NA	1.R 2.S 3.C 4.NA	1.R 2.S 3.C 4.NA	1.R 2.S 3.C 4.NA		1.Direct Specimen 2. Culture Positive	1.Detected 2.Not Detected		1.R 2.S 3.I			1.R 2.S 3.I			1.R 2.S 3.I			1.R 2.S 3.I	1.R 2.S 3.I	1.R 2.S 3.I	1.R 2.S 3.I
									1.S 2.M 3.B 4.O	1.S 2.M 3.B 4.O	1.Neg 2. Scanty 3.1+ 4.2+ 5.3+	1.Neg 2. Scanty 3.1+ 4.2+ 5.3+	1.MTB ND 2.Rif Sen. 3.Rif Res. 4.Rif Ind.	1.MTB ND 2.Rif Sen. 3.Rif Res. 4.Rif Ind.					1.MTBC 2. NTM 3. Neg. 4. Cont.	1.Solid 2. Liquid			1.R 2.S 3.C 4.NA	1.R 2.S 3.C 4.NA	1.R 2.S 3.C 4.NA	1.R 2.S 3.C 4.NA	1.R 2.S 3.C 4.NA	1.R 2.S 3.C 4.NA	1.R 2.S 3.C 4.NA	1.R 2.S 3.C 4.NA	1.R 2.S 3.C 4.NA	1.R 2.S 3.C 4.NA	1.R 2.S 3.C 4.NA	1.R 2.S 3.C 4.NA	1.R 2.S 3.C 4.NA		1.Direct Specimen 2. Culture Positive	1.Detected 2.Not Detected		1.R 2.S 3.I			1.R 2.S 3.I			1.R 2.S 3.I			1.R 2.S 3.I	1.R 2.S 3.I	1.R 2.S 3.I	1.R 2.S 3.I
									1.S 2.M 3.B 4.O	1.S 2.M 3.B 4.O	1.Neg 2. Scanty 3.1+ 4.2+ 5.3+	1.Neg 2. Scanty 3.1+ 4.2+ 5.3+	1.MTB ND 2.Rif Sen. 3.Rif Res. 4.Rif Ind.	1.MTB ND 2.Rif Sen. 3.Rif Res. 4.Rif Ind.					1.MTBC 2. NTM 3. Neg. 4. Cont.	1.Solid 2. Liquid			1.R 2.S 3.C 4.NA	1.R 2.S 3.C 4.NA	1.R 2.S 3.C 4.NA	1.R 2.S 3.C 4.NA	1.R 2.S 3.C 4.NA	1.R 2.S 3.C 4.NA	1.R 2.S 3.C 4.NA	1.R 2.S 3.C 4.NA	1.R 2.S 3.C 4.NA	1.R 2.S 3.C 4.NA	1.R 2.S 3.C 4.NA	1.R 2.S 3.C 4.NA	1.R 2.S 3.C 4.NA		1.Direct Specimen 2. Culture Positive	1.Detected 2.Not Detected		1.R 2.S 3.I			1.R 2.S 3.I			1.R 2.S 3.I			1.R 2.S 3.I	1.R 2.S 3.I	1.R 2.S 3.I	1.R 2.S 3.I

Workbook last saved: Just now

TB Diagnostic Center DRS Register

S.N.	Patient Name	Contact details	Smear Microscopy details as recorded in CIF*			Xpert MTB/RIF details as recorded in CIF*			Patient enrolled for DRS?	DRS Enrolment date** *(DD/MM/YYYY)*	Date of shipment of samples to Central Laboratory *(DD/MM/YYYY)*
			Date of test *(DD/MM/YYYY)*	Test results	Name of Microscopy centre	Date of test *(DD/MM/YYYY)*	Test result	Name of GeneXpert centre	*If Patient was enrolled for DRS, attach DRS Patient ID barcode; otherwise write reasons for not enrolling*		

** Microscopy and/or Xpert MTB/RIF results must be also recorded in the Clinical Information Form (CIF) of each enrolled patient.*
