# Supplementary material for: Drug-resistant tuberculosis in Nepal: the sixth national prevalence drug-resistant survey, 2024
Source: Infect Dis Poverty. 2026 Oct 13;15:109. doi: 10.1186/s40249-026-01503-z (PMC13660163; doi:10.1186/s40249-026-01503-z)
Supplement: Supplementary file 3 [file 40249_2026_1503_MOESM3_ESM.pdf]

Survey Team Training

Before field implementation, a structured training program was designed for all personnel involved in the survey. The training sessions were tailored to the roles and responsibilities of different groups and conducted at both central and field levels. The key components of the training were as follows:

- **Central-Level Training**: Conducted by the NTCC, focusing on specimen collection, enrolment protocols, and logistics.

- **Field-Level Training**: Delivered by the Outsource Agency under the supervision of the NTCC survey team, emphasizing specimen handling, patient enrolment, and record-keeping.
- **Specialized Laboratory Training**: Organized by the SRL Gauting, Germany for personnel from central laboratories (NTRL/NTCC and GENETUP) to standardize laboratory procedures.

The training content was developed based on Standard Operating Procedures (SOPs) and Terms of Reference (ToR) to ensure consistency and adherence to guidelines.

Patient Enrolment and Specimen Collection

Eligible patients (new and previously treated pulmonary TB cases) were enrolled at selected peripheral diagnostic centers. The key procedures were as follows:

- **Patient Enrolment**: Informed consent and assent were obtained from each patient. A face-to-face interview was conducted to complete a CIF, documenting the patient's clinical history, and confirming eligibility.
- **Specimen Collection**: Two sputum samples (spot and morning) were collected from each patient. The samples were carefully labelled with unique barcoded IDs to ensure proper identification and tracking.
- **Data Recording**: Each enrolled patient was registered in the TB Diagnostic Centre DRS Register (Annex-V) and the DRS Shipment Register. This ensured comprehensive documentation and traceability throughout the study process.

Sample Transport

Collected sputum samples were transported to central laboratories under strict cold-chain conditions. The key steps in the transport process were as follows:

- **Packaging**: Samples were secured using triple-layer packaging within cold boxes containing ice packs to maintain the required temperature.
- **Shipment**: Daily transport was arranged to ensure samples reached the laboratory within three days of collection for timely processing.
- **Documentation**: Each batch was accompanied by consent forms, clinical information, and shipment registers to ensure proper traceability.

The specimen transport process complied with International Air Transport Association guidelines for infectious substances.

Laboratory Algorithm Overview

The survey's laboratory algorithm followed a systematic approach to diagnose and profile drug-resistant TB. The key steps were as follows:

- Sample collection and transportation: Sputum specimens were collected from eligible pulmonary TB patients and transported to central laboratories (NTRL/NTCC or GENETUP).
- Diagnostic Testing: Samples were tested using microscopy, Xpert MTB/RIF, and both phenotypic and genotypic DST.
- Drug Resistance Profiling: Further testing was conducted on RR-TB cases to assess resistance to second-line and new/repurposed drugs. Additionally, all confirmed MTBC samples underwent levofloxacin susceptibility testing.

Viable isolates were cryopreserved for future analyses and quality control purposes. Figure 1 presents the detailed laboratory algorithm.

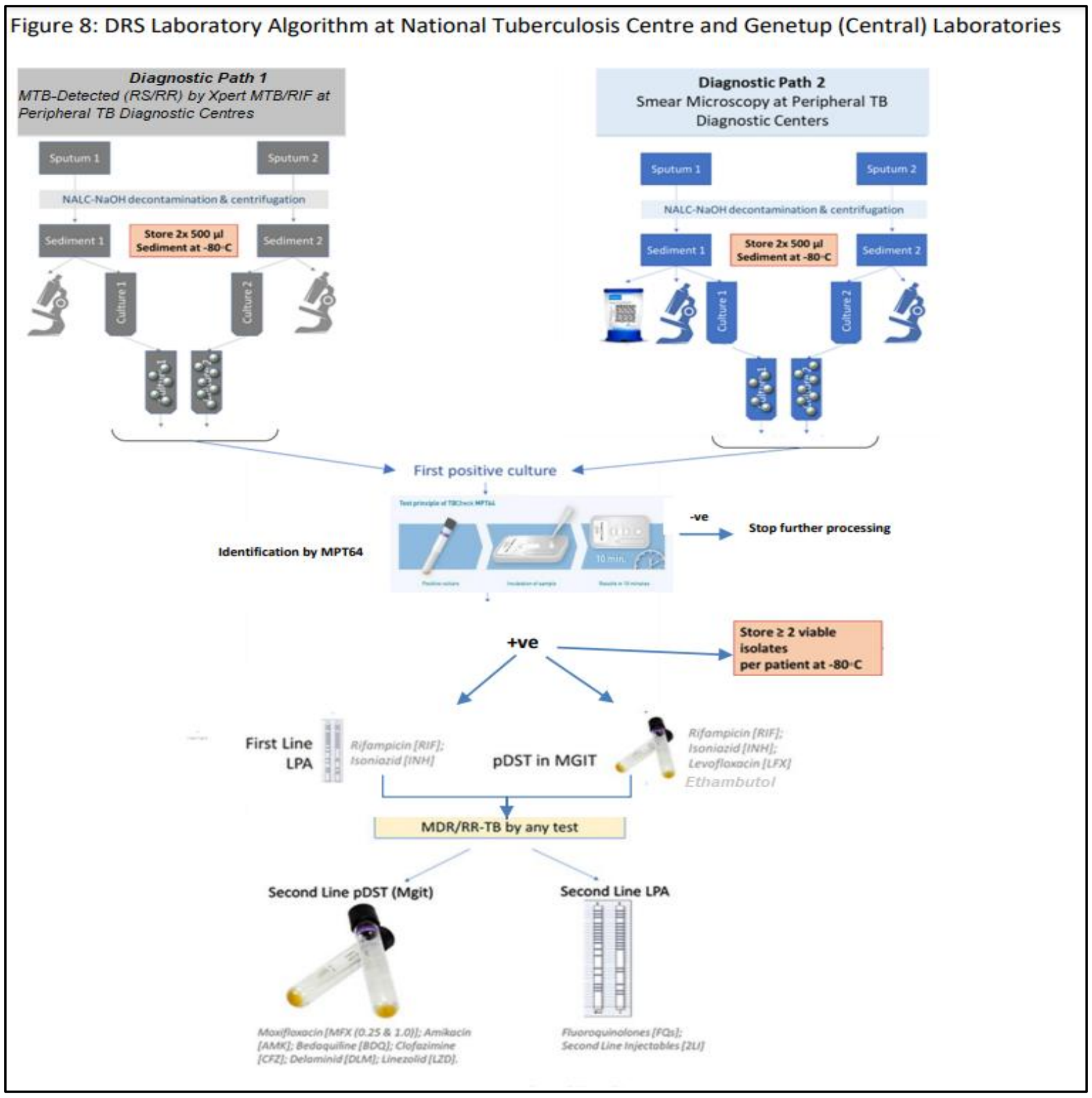

Figure 1: Laboratory algorithm at central laboratories

Sample Processing and Testing

Upon receipt at central laboratories (NTRL/NTCC and GENETUP) the following procedures were conducted:

- **Initial Testing**: Samples underwent decontamination, smear microscopy, and culture on Lowenstein-Jensen (LJ) media.
- **Diagnostic Pathways**: Samples were stratified into two pathways based on prior Xpert MTB/RIF testing results.
- **Drug Susceptibility Testing**: Phenotypic and genotypic DST were conducted to determine resistance profiles for both first- and second-line anti-TB drugs.
- **Quality Control**: Isolates were preserved at -80°C for future re-testing and three levels of EQA

All laboratory activities were guided by detailed SOPs to ensure consistency and reliability.

Quality Assurance and Quality Control:

Quality Assurance in Protocol and Methods

The survey protocol strictly adhered to the WHO guidelines (6), employing nationally representative sampling with randomization to minimize selection bias. Feedback from national and international experts was incorporated to ensure the protocol's standardization, usability, and comparability with global surveys.

Quality in Organization, Management, and Budget

The survey was managed through dedicated committees (see Annex-VI) to ensure accountability and smooth implementation. WHO provided external quality assurance. Moreover, dedicated NTCC staff, WHO-Nepal Staff, and staff for the survey through SCI/GF support and other technical partners provided internal quality assurance under the guidance of the working committee. Fieldwork was outsourced to an experienced agency, and full-time human resources were recruited to ensure effective execution. The survey was adequately funded through a combination of domestic resources and donor contributions. Two central laboratories were utilized, with one designated as a backup to maintain operational efficiency and SRL Gauting provided external quality assurance of the central laboratory for the survey.

Quality in Implementation

Fieldwork implementation

Fieldwork was conducted by an outsourced agency, with rigorous training provided by the NTCC. Monitoring and coaching were conducted by committee members and Liaison and Quality Assurance Officers (LQAOs). Each cluster was monitored at least twice, with activities guided by systematic checklists. Regular meetings with WHO and SRL teams were held to address initial challenges and ensure adherence to DRS SOPs. Key indicators were tracked using real-time dashboards to maintain quality and monitor progress effectively.

Central lab work implementation

Central laboratory activities were closely supervised by the Lab Chief, with regular reports reviewed during committee meetings to ensure accountability and progress. SRL Gauting provided necessary feedback and support on the third level of EQA.

Quality Assurance of Laboratory Procedures

Internal quality assurance

Regular Internal quality controls (IQC) were performed for all laboratory procedures in both Central Laboratories.

External quality assurance (EQA)

Three EQA levels were implemented:

First Level EQA: Retesting of isolates with discrepancies or unexpected resistance patterns was conducted at the alternate central laboratory (NTRL/NTCC to GENETUP and vice-versa) within Nepal.

Second Level EQA: A random 10% of rifampicin-susceptible isolates, along with all rifampicin-resistant isolates were re-tested at the alternate central laboratory (NTRL/NTCC to GENETUP and vice-versa).

Third Level EQA: Isolates with unresolved discrepancies from the first-level EQA or unexpected resistance patterns not confirmed locally were sent to the SRL Gauting, Germany for the final result.

This robust quality assurance framework ensured the reliability and accuracy of the survey findings.
